# The potential for soybean to diversify the production of plant-based protein in the UK

**DOI:** 10.1016/j.scitotenv.2020.144903

**Published:** 2021-05-01

**Authors:** Kevin Coleman, Andrew P. Whitmore, Kirsty L. Hassall, Ian Shield, Mikhail A. Semenov, Achim Dobermann, Yoann Bourhis, Aryena Eskandary, Alice E. Milne

**Affiliations:** a Sustainable Agriculture Sciences Department, Rothamsted Research, Harpenden, Hertfordshire AL5 2JQ, UK; b Computational and Analytical Sciences Department, Rothamsted Research, Harpenden, Hertfordshire AL5 2JQ, UK; c Plant Sciences Department, Rothamsted Research, Harpenden, Hertfordshire AL5 2JQ, UK; d Rothamsted Research, Harpenden, Hertfordshire AL5 2JQ, UK.

**Keywords:** Rothamsted Landscape Model, Soil processes, Nutrient flow, Soya bean, Agriculture, Future climate

## Abstract

Soybean (*Glycine max*) offers an important source of plant-based protein. Currently much of Europe's soybean is imported, but there are strong economic and agronomic arguments for boosting local production. Soybean is grown in central and eastern Europe but is less favoured in the North due to climate. We conducted field trials across three seasons and two sites in the UK to test the viability of early-maturing soybean varieties and used the data from these trials to calibrate and validate the Rothamsted Landscape Model. Once validated, the model was used to predict the probability soybean would mature and the associated yield for 26 sites across the UK based on weather data under current, near-future (2041–60) and far-future (2081–2100) climate. Two representative concentration pathways, a midrange mitigation scenario (RCP4.5) and a high emission scenario (RCP8.5) were also explored. Our analysis revealed that under current climate early maturing varieties will mature in the south of the UK, but the probability of failure increases with latitude. Of the 26 sites considered, only at one did soybean mature for every realisation. Predicted expected yields ranged between 1.39 t ha^−1^ and 1.95 t ha^−1^ across sites. Under climate change these varieties are likely to mature as far north as southern Scotland. With greater levels of CO_2,_ yield is predicted to increase by as much as 0.5 t ha^−1^ at some sites in the far future, but this is tempered by other effects of climate change meaning that for most sites no meaningful increase in yield is expected. We conclude that soybean is likely to be a viable crop in the UK and for similar climates at similar latitudes in Northern Europe in the future but that for yields to be economically attractive for local markets, varieties must be chosen to align with the growing season.

## Highlights

• Data on novel soybean varieties was used to calibrate and validate the Rothamsted Landscape Model.
• Simulations were run for 26 UK sites using current and future climate scenarios.
• Under current climate early-maturing varieties matured in the south of the UK.
• Under climate change soybean is predicted to mature as far north as Scotland.
• No meaningful increases in yield are predicted under climate change.

## Graphical abstract

**Figure:**
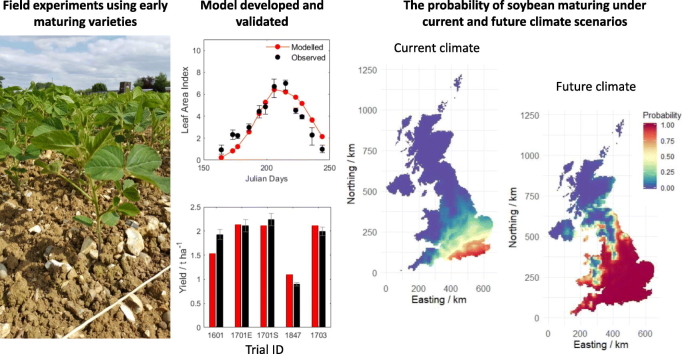

## 1 Introduction

In 2019 the Eat-Lancet commission published a report that established clear scientific targets to guide transformation to a healthier more sustainable food system (Willett et al., 2019). At the top of the list of strategies to achieve this urgently needed change is a call to increase the consumption of plant-based foods and substantially reduce the consumption of animal source foods. This accords with the research of others who have quantified the relative inefficiencies of meat-based food compared with plant-based (Reijnders and Soret, 2003; Sabate and Soret, 2014; Springmann et al., 2016). Tessari et al. (2016) countered the argument that plant-based proteins were less environmentally damaging than animal-based proteins by comparing production based on the delivery of essential amino acids. They demonstrate that animal production has a similar environmental impact to plant production on an essential amino acid basis, with the exception for soybean (*Glycine max*), which has a significantly smaller impact.

Globally, soybean is an important source of plant-based protein, with a percentage of crude protein larger than many other legumes or pulses in commercial production (Cheng et al., 2019). Total soya consumption in the UK is estimated to be 3.8 million t, including soya beans and meal, but also 0.7 million t imported as soya embedded in other product (efeca, 2018). Currently much of Europe's soybean is imported from the United States and South America (European Commission, 2019), with only modest amounts of it grown in Europe itself, particularly in the southeastern and eastern regions of the European continent, as locally produced, non-GM soybean for feed and oil, or for premium markets such as organic food and fresh vegetables (IDH and IUCN NL, 2019). The EU non-GM soy market accounts for around 15% of the total feedgrade market and growing consumer concerns over environmental and animal welfare issues are expected to further segment the livestock feed market between conventional and premium feed. Hence, the search for alternative protein sources in Europe is driven by a desire to increase self-sufficiency in these market niches, which enable European soybean farmers to charge premiums of €80 to €120 per t of non-GM soybeans, with organic soy earning double this premium (Curtis et al., 2006).

Besides such economic incentives, there are other reasons for boosting more local production. Direct consumption of soybean by humans is likely to rise due to shifts towards more plant-based diets (FarmingUK, 2018; Román et al., 2017; Tuorila and Hartmann, 2020). Moreover, European agriculture is in dire need for diversification and would greatly benefit from an economically viable, N-fixing legume that breaks the pest, competitor or disease cycles in the main cash crops that dominate current rotations. New agricultural policies in the EU as well as in the UK will likely stimulate agronomic measures that diversify cropping and/or benefit soil health and other ecosystem functions.

Soybean crops are grown in cold-temperate regions, such as the USA and Canada, as well as sub-tropical and tropical regions. Temperatures between 22 and 35 °C are best suited for growth. If average temperatures fall below this then there is a delay in development lowering the chances of the crop reaching maturity. This is an issue for growing soybeans in Northern Europe. Despite this, soybean has been grown commercially in the UK since at least the late 1990's but take up has been limited because the available varieties were not well suited to the UK climate and there were difficulties in harvesting. Recent advances in breeding mean that there are now more varieties that mature earlier (which is essential for the UK's colder, wetter climate) and have a canopy architecture that makes them easier to harvest. This means soybean could become a viable plant-based alternative source of protein for UK production systems.

As well as providing an alternative to animal-based protein (being relatively rich in the amino-acids Lysine and Methionine unlike most other legumes currently grown in Europe) there are several other benefits to growing soybeans in the UK. First, as a leguminous crop soybean can fix nitrogen reducing the need for fertilizer and increasing system-level N use efficiency. Second, with increasing resistance of weeds, slugs, insect-pests and diseases to chemical control agents, and the loss of active ingredients due to more stringent legislation, diverse crop rotation, including a spring sown protein crop such as soybean is becoming of increasing agronomic interest to UK farming. A key question facing farmers, however, is what is the likelihood that the crop will grow successfully, and can this crop be a profitable part of a diverse crop rotation now and in the future? Research trials can help answer these questions in part, but they are both expensive and time consuming and questions related to the effects of climate change become infeasible to test: therefore, we turn to models.

In this study, we set out to determine the spatial extent over which soybean is a viable crop in the UK based on the current climate, and to determine how this is likely to alter under climate change. For this we consider both the probability that early maturing varieties of soybean will mature, and the yield that could be expected. To achieve this we used data from field trials designed to test the viability of growing earlier maturing varieties of soybean in the UK to calibrate and validate the crop model in the Rothamsted Landscape Model (Coleman et al., 2017) for soybean. Once the model was validated, we used it with simulated weather data based on current and future climates for 26 sites across the UK to determine the probability that soybean crops would mature, and how this is affected by location and climate change.

## 2 Methodology

### 2.1 Soybean trials

Between 2016 and 2018, a total of six field trials were carried out at Rothamsted Research's experimental farms located in Harpenden, Hertfordshire, UK (51° 48′ N, 0° 21′ W), and Brooms Barn, near Bury St Edmunds Suffolk, UK (52° 16′ N, 0° 34′ E) to test the viability of early maturing soybean varieties under UK conditions. At each trial between two and twelve advanced breeding lines or varieties that had been developed in North America were grown in randomised replicated plot designs with variety as the treatment factor (S.I. Table S7). In 2018 two European varieties were also tested at each site (full details are given in S.I. Table S8). The materials were chosen in consultation with breeders working in the more northern growing areas of North America, where the temperatures are lower, and the day-length is similar to that in the UK. The maturity groupings of each variety tested ranged between 000 and 0 and are given in Table S8 (Song et al., 2019) For trial 1701 sowing time was also used as a treatments factor (see Table 1). No inorganic fertilizer was applied to the experiments, but the soybean seed was inoculated with Bradyrhizobium japonicum (Legume Technology, Nottinghamshire, UK). Standard herbicide and molluscicide programmes were applied to control weeds and slugs, and some bird protection was required. Little disease was detected. The soil at Rothamsted is described as silty clay loam (Batcombe series) by Avery and Catt (1991) and Aquic (or Typic) Paleudalf (Soil Survey Staff, 1999). The soil at Broom's Barn is a Sandy Loam belonging to the Moulton and Ashley Variant series. Both sites are research farms with closely monitored soil physical condition and nutritional status. As such, we found no notable nutrient deficiencies or soil physical impediments in the soil.

**Table 1:** Details of the six trials at Harpenden (H) and Brooms Barn (B). The trials used as our validation set are marked by *.

Trial ID	Year	Site	Field name	Number of varieties grown	Sowing dates	Seed rates/seeds m^−2^	Harvested^a^
1601	2016	H	Great Field 4	9	27th April	45	22nd September
1701	2017	H	Great Knott 3	2	3rd and 28th April	60	4th October
1847	2018	H	Great Knott 3	6	25th April	60	13th November
1703	2017	B	Dun Holme	12	27th April	60	17th October
1702	2017*	H	Fosters	12	28th April	60	4th October
1848	2018*	B	Marl Pit	6	10th May	60	19th September

a Some trials were harvested over a number of days for practical reasons and the date given is the earliest of the recorded dates.

Soybean yields were measured on each of the six trials (see Table 1). The nitrogen (N) in the seed was measured in two trials (trial references 1702 and 1703). Leaf area index (LAI) was measured at two trials (trial references 1701 and 1702). To ensure we had both LAI and seed N measures in both the validation and calibration sets and to maximise site and season diversity in both sets, we chose to use experiments 1601, 1701, 1703 and 1847 for our calibration set and 1702, and 1848 for our validation set.

### 2.2 The soybean model

The Rothamsted Landscape Model (Coleman et al., 2017) is a daily process-based model that simulates soil processes (including soil organic matter, soil nutrient and water dynamics), livestock production, crop growth and yield of cereals (wheat, barley, and oats), oilseed rape, field beans, sugar beet, forage maize, potato, onions and grass. The crop model, which is based on the LINTUL 5 model (Wolf, 2012), uses daily weather variables to predict canopy development and resource accumulation. The weather data required to run the model is minimum and maximum temperature, rainfall, solar radiation, vapour pressure and windspeed. The model can be run as a point scale model or in a spatially explicit fashion with adjacent pieces of land (fields or watercourses) linked to simulate spatial movement of water and nutrients. The model components are based on well-established existing models such as RothC (Coleman and Jenkinson, 2014), LINTUL (Wolf, 2012), SUCROS (van Laar et al., 1997), and Century (Parton et al., 1994) as described in Coleman et al. (2017), and water movement as described by Addiscott and Whitmore (1991) and Van Ittersum et al. (2003).

The crop model (which is based on LINTUL, Wolf (2012)) is a generic plant growth model, which has a bespoke parameterisation for each crop modelled. It uses a light use efficiency (LUE, g dry matter MJ^−1^) based approach to calculate biomass production (Monteith, 1990; Monteith and Moss, 1977). The rate of biomass (*B*_crop_) produced each day is given by

(1) $$\frac{dB_{\text{crop}}}{\mathrm{dt}}=Q\;\varepsilon \;W_{\mathrm{rf}}\;N_{\mathrm{NI}}\;P_{\mathrm{NI}}\;F_{{\mathrm{CO}}_{2}}$$

where *Q* is the intercepted PAR (MJ PAR m^−2^ surface area) which depends on the solar radiation and canopy leaf area, *ε* is the crop specific LUE, *W*_rf_ is the transpiration reduction factor, *N*_NI_ and *P*_NI_ are nitrogen and phosphorus nutrition indices, which range from zero to one, *F*_CO2_ is a CO_2_ factor which allows dry matter production to change according to

(2) $$F_{{\mathrm{CO}}_{2}}=1.52-1.74\;\left(0.9966^{{\mathrm{CO}}_{2}}\right)$$

where CO_2_ is the atmospheric CO_2_ in ppm. This function is based on that in Wolf (2012). The biomass formed is partitioned between roots, stem, leaves and storage organs based on the development stage (D) which starts from zero at germination and finishes at a value of two which represents maturity (Boons-Prins et al., 1993; Wolf, 2012). Development stages accumulate as a function of photo-vernal-thermal time (as described in Wolf (2012) and Weir et al. (1984)).

The uptake of plant nutrients (N and P) is determined by the crop demand and the supply of these nutrients by soil. The total nutrient demand of the crop is the sum of the nutrient demand from its individual organs, i.e. roots, stems and leaves excluding storage organs, for which nutrient demand is met by translocation from the other organs. Note that in our version of this model, translocation from roots follows similar dynamics to that of stem and leaves to avoid cases where the stem and leaves become depleted of N whilst large amounts remain in the roots, in all cases the translocation rate was set to 1. Nutrient demand of the individual organs is calculated as the difference between maximum and actual organ nutrient contents. The maximum nutrient content is defined as a function of canopy development stage. For most crops including soybean, the total nutrient uptake of the crop takes place before anthesis. Sub-optimal nutrient availability in the soil leads to nutrient stress in the crop. A detailed description of crop N dynamics is reported by Shibu et al. (2010). Further details for N and P are given in Coleman et al. (2017).

To model soybeans and their interaction with soil nutrient cycling, we included processes related to daily biological N fixation (*N*_*BNF*_). For this, we adopted the model described in Bouniols et al. (1991) and Williams et al. (1989). Biological N fixation (*N*_*BNF*_) is assumed to be

(3) $$N_{\mathrm{BNF}}=\min\left[N_{\mathrm{Dem}}\;f\left(D,w,N_{\mathrm{SMN}}\right),B_{\mathrm{Max}}\right]$$

where *B*_Max_ is the maximum that *N*_*BNF*_ per day and assumed to take the value 6.0 (following LINTUL (Wolf, 2012)). The variable *N*_Dem_ is the total N demand of the crop, *f*(*D*, *w*, *N*_*SMN*_) is a function of crop development stage (*D*), soil water (*w*), and soil mineral-N content (*N*_*SMN*_), given by

(4) $$f\left(D,w,N_{\mathrm{SMN}}\right)=g_{\mathrm{DVS}}\left(D\right)\min\left[g_{w}\left(w\right),g_{\mathrm{SMN}}\left(N_{\mathrm{SMN}}\right)\right]$$

The functions *g*_*DVS*_(*D*), *g*_*w*_(*w*) and *g*_*SMN*_(*N*_*SMN*_) are scaling factors; *g*_*DVS*_(*D*) rises linearly from zero at *D* = 0.2 to reach a maximum of one at *D* = 0.6. It then reduces linearly from a value of one at *D* = 1.2 to zero at *D* = 1.6. Outside of the range *D* = (0.2, 1.6) it is zero (see Bouniols et al. (1991) noting that their development stages are scaled by a factor of 0.5 compared with ours). The function *g*_*w*_(*w*) is zero when *w* is less than 0.45 of the difference between field capacity and wilting point and rises linearly to a maximum of one at field capacity. The function *g*_*SMN*_(*N*_*SMN*_) takes a value of 1 when the average *N*_*SMN*_ in the rooting depth of the soil is less than 100 kg-N ha^−1^ m^−1^, falling linearly to zero at 300 kg-N ha^−1^ m^−1^. We note that in the model our soil profile is assumed to be 1-m in depth (see Coleman et al., 2017).

The biological N fixed each day (BNF) is added to the N in the root, stem and leaves. The proportional split is based on the N already in each part of the plant. For example, the addition N partitioned to the leaves (*η*_leaf_) is given by

(5) $$\eta_{\text{leaf}}=N_{\mathrm{BNF}}\frac{N_{\text{leaf}}}{N_{\text{leaf}}+N_{\text{stem}}+N_{\text{root}}}$$

where *N*_leaf_, *N*_stem_, and *N*_root_ are the amounts of *N* in the leaf, stem and root prior to the daily addition of N from BNF. Santachiara et al. (2018) found no evidence to suggest that BNF constitutes a net extra energy cost to soybean crop in terms of growth or yield. Therefore, similar to Sinclair et al. (2003), we assume none in our model.

### 2.3 Model parameterisation and calibration

We used the soybean model parameter values reported in Wolf (2012) for our model. We noted, however, that the maximum N in the seed from trial 1703 experiments was larger than the value allowed by the existing parameterisation and so we increased the value of the parameter defining this from 5.6% to 7.35%, which is the maximum seed N content of our calibration trial 1703. In addition, we expected the new varieties to have earlier flowering dates and a different canopy structure than those reported in Wolf (2012) and we noted that other LINTUL-based models of soybean (Corrêa, 2008) proposed smaller values for light use efficiency (LUE) and greater values for specific leaf area than those reported in the original LINTUL model. Therefore, we recalibrated the LUE, specific leaf area, anthesis and maturity parameters using the data from our experiments. Our aim was to minimise the root mean squared error (RMSE) between measured and modelled values of LAI and yield.

### 2.4 Climate scenarios

**Fig. 1:**
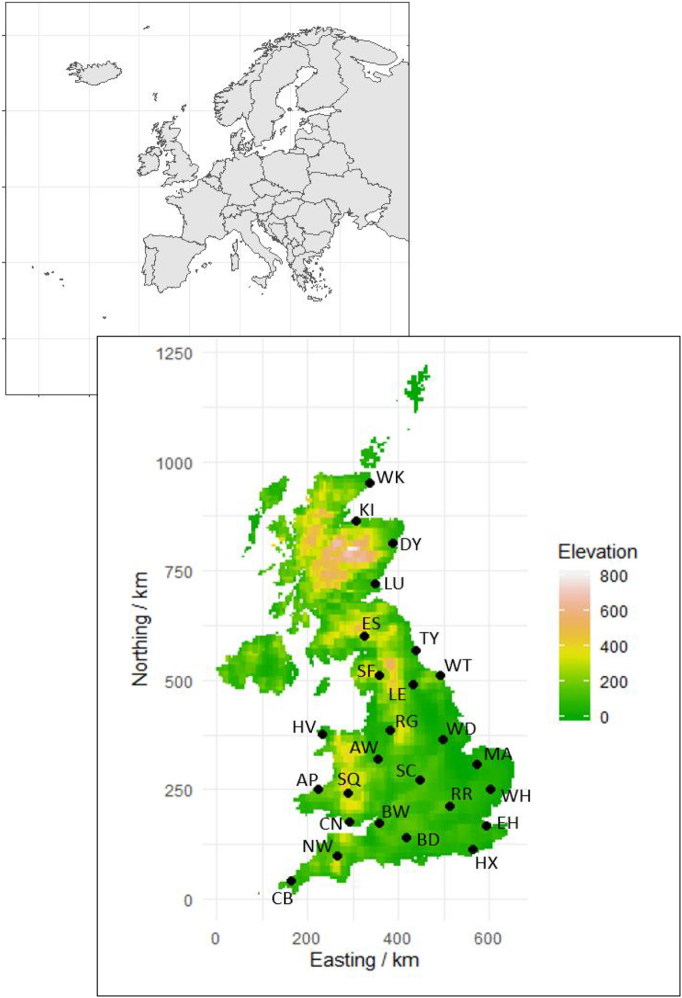
A map of the UK showing the location of the climate stations (black dots) that were used in the simulations. The map was produced using the R software. We use OSGB Cartesian co-ordinates as measures of easterly and northerly distance. The location of the UK in Europe is shown the in inset pane.

We ran the simulation model with weather data generated from current climate (1980–2010), near-future climate scenarios (2041–2060) and far-future climate scenarios (2081–2100) for 26 sites across the UK (see Fig. 1). The current climate was based on daily observed weather data during 1981–2010. The summary statistics for temperature and precipitation at each site are listed in the S.I. Table S10. The future weather scenarios were based on climate projections from 18 global climate models (GCMs) from the multi-model ensemble used in IPCC Assessment Report 5 (AR5) (Taylor et al., 2012), two representative concentration pathways (RCPs), a midrange mitigation scenario (RCP4.5) and a high emission scenario (RCP8.5) (van Vuuren et al., 2011), and two future points in time (near 2041–60 and far 2081–2100 future). This resulted in four future climate sets which we refer to as (i) near-future-RCP4.5, (ii) near-future-RCP8.5, (iii) far-future-RCP4.5 and (iv) far-future-RCP8.5. To generate the local-scale future daily weather scenarios for each set, we used the LARS-WG weather generator (Semenov et al., 2010), a stochastic weather generator used in many recent European climate change impact and risk assessments (Trnka et al., 2015; Trnka et al., 2014; Vanuytrecht et al., 2014). For further details see (Semenov and Stratonovitch, 2015) and Harkness et al. (2020). Vapour pressure and windspeed, not generated by the LARS-WG, were estimated using methods described by the FAO (Allen et al., 1998).

Due to the coarse spatial and temporal resolution of GCMs and large uncertainties in the model outputs, it is not appropriate to use daily output from GCMs in combination with nonlinear process-based models when analysing impacts of changes in climatic variability and extreme weather events (Semenov et al., 2010). Therefore, for each of our 26 sites, we downscaled the climate projections from GCMs to local-scale daily climate scenarios by using LARS-WG, a stochastic weather generator (Semenov and Stratonovitch, 2010). LARS-WG downscales the projections from the GCMs to a local scale, incorporating changes in the mean climate, climatic variability and extreme events derived from the GCMs, by modifying the statistical distributions of the weather variables (Semenov, 2007).

For each [site] × [climate set] × [GCM], future synthetic daily weather data (300 realisations of single weather years) were generated by the LARS-WG weather generator based on changes in distributions of climate variables derived from each GCM and emissions scenario. The CO_2_ concentration for each climate sets listed in Table 2, along with the CO_2_ concentration assumed for the current climate set. To understand the relative effects of climate change and increases in CO_2_, we also ran the model with current climate weather data and the CO_2_ concentration associated with far-future-RCP8.5 (i.e. 844). The model was run for each year and the date of soybean maturity and yield were recorded. For soybean to be a viable crop it must mature early enough to not disrupt the sowing of the next crop in the rotation and also to avoid weather conditions unfavourable for drying the crop in the field, risking difficult harvest conditions and expensive artificial drying of the crop. On the advice of our agronomist (an author of this paper) we decided on a cut-off date of the 1st Oct with soybean crops maturing before this date deemed viable. Based on this, the variables of interest in our study are the probability that soybean will mature before 1st Oct and the yield. It should be noted, however, that this is a conservative cut-off date, i.e. in many years harvest of soybean and sowing of winter crops could still be feasible later in October.

**Table 2:** Concentrations of CO_2_ (ppm) for current, RCP4.5 and RCP8.5. The current values are based on measurements from 2017 and the future on those reported in Harkness et al. (2020).

	Current	RCP 4.5	RCP 8.5
2017	405		
2041–2060		487	541
2081–2100		533	844

### 2.5 Statistical analysis

For each Site by Climate combination, the probability of maturity was calculated as the proportion of simulations (out of 300) that resulted in maturity before the 1st October. Under future climate scenarios, this was averaged over the 18 GCMs,

(6) $$P\left(\text{Maturity}\right)=\left\{\begin{array}{c} \frac{\#\text{Mature}}{300},\text{if current scenario}\\ \frac{\frac{1}{18}\sum_{\mathrm{GCM}=1}^{18}\#{\text{Mature}}_{\mathrm{GCM}}\;}{300},\text{if future scenario} \end{array}\right)$$

For many Climate × Site combinations, the probability of maturity is estimated at the boundaries of the [0, 1] interval. Thus, for consistency, confidence intervals for the probability of maturity were obtained using the Clopper-Pearson (Clopper and Pearson, 1934) approach with interval defined by,

(7) $$\text{Beta}\left(\frac{\alpha }{2};x,n-x+1\right),\text{Beta}\left(1-\frac{\alpha }{2};x+1,n-x\right),$$

where *x* is the numerator in Eq. (6), *n* is the denominator and Beta (*p*, *a*, *b*) is the *p*th quantile from a beta distribution with parameters *a* and *b*.

Yield was analysed only where maturity occurred, a total of 419,386 simulation runs. The following linear model was fitted

(8) $${\text{Yield}}_{i}=\left({\text{Climate}}_{i}\backslash \left({\mathrm{RCP}}_{i}*{\text{Period}}_{i}*{\mathrm{GCM}}_{i}+\text{AtmCO}2_{i}\right)\right)*{\text{Site}}_{i}+\varepsilon_{i}$$

where *ε*_*i*_ are iid Normal random variables. The factor Climate has two levels; Climate and Future. Levels of the factors RCP, Period and GCM vary only in Future climate scenarios, whilst levels of AtmCO2 vary only in Current climate scenarios. High levels of imbalance in the number maturing results in unequal numbers of yield observations across the different factors. Consequently, results are analysed through both the marginal (respecting marginality) and conditional F-statistics.

Clopper-Pearson intervals were calculated in the R software environment (RStudio Team, 2020). The linear model for Yield was fitted in Genstat 20th edition (VSN International, 2019).

Maturity is based on climatic data and day length, and so longitude, latitude and elevation are plausible covariates to support spatial prediction. Therefore, to support spatial prediction (mapping) of the probability of maturity we fitted linear models to the logit of the probability of soybean maturing with these covariates as explanatory variables, and then used these covariates to predict the probability of maturity across the UK.

## 3 Results

### 3.1 Soybean trials

The soybean crop successfully matured in all field trials conducted. Yields ranged between 0.4 t ha^−1^ in 2018 to 2.9 t ha^−1^ in 2017 with an average of 1.7 t ha^−1^ (Table 3 and Supplementary information). In general, yields at Brooms Barn were greater than those at Harpenden. In all experiments, varietal performance differed significantly in terms of yield (Table S8).

**Table 3:** The summary statistics for soybean seed yield for each trial. The trials used as for validation are marked by *.

Trial ID	Sowing time	Seed yield/t ha^−1^ at 14% moisture content					
		Mean	Variance	Number of plots	Standard error	Min	Max
1601	Standard	1.929	0.292	27	0.104	0.860	2.822
1701	Early	2.113	0.0951	8	0.109	1.645	2.527
1701	Standard	2.235	0.182	8	0.151	1.786	2.805
1703	Standard	1.992	0.271	33	0.0907	0.491	2.888
1847	Standard	0.898	0.0390	30	0.0360	0.392	1.325
1702*	Standard	1.714	0.178	33	0.0734	0.808	2.490
1848*	Standard	1.283	0.0729	30	0.0493	0.639	1.696

Across the 2016 and 2017 variety trials the highest yielding cultivars gave moderate yields with the means over replicate plots having maximum of 2.72, 2.34 and 2.61 t ha^−1^. Yields in 2018 were substantially lower with a maximum of 1.08 t ha^−1^ (Harpenden) and 1.61 t ha^−1^ (Brooms Barn) primarily due to the exceptionally dry weather during the months of June and July (see Supplementary information Tables S2–6) which affected the soil moisture. Despite the reasonable water holding capacity of the silty clay loam soil at Harpenden it is not unusual for later spring sown crops to suffer from drought as rooting fails to extend sufficiently rapidly to maintain water supply to the plant.

Analysis of trial 1701 showed significant differences in yield according to sowing date (F_1,3_ = 24.15, p = 0.016) with late drilling yielding an average of 0.18 t ha^−1^ more. Given that variety was not accounted for in our model we calibrated our simulations to the mean values of yield and seed N across varieties for each site, season and sowing time (Table 3, Table 4). A complete analysis of the trials data for all years is given in the Supplementary information.

**Table 4:** The summary statistics for soybean seed N. The trials used for validation are marked by *.

Trial ID	Seed N/%					
	Mean	Variance	Number of plots	Standard error	Min	Max
1702*	6.600	0.121	33	0.0607	5.912	7.1
1703	6.670	0.133	33	0.0608	6.013	7.345

There was no consistent response of variety between seasons, and this is disappointing from the point of view of selecting well adapted genetics for UK agriculture. It was noted that the rhizobium applied to the seeds in 2016 was poor quality (the peat-based carrier had dried out) resulting in few root nodules and low seed nitrogen contents (data not presented). The seed of two of our varieties sown in 2016 was poor quality and this was reflected in low plant counts (see Supplementary information Table S8, varieties Canada 4 and 6). Fresh seed was sown in 2017 and a new, liquid, formulation of rhizobium was applied.

### 3.2 The soybean model calibration and validation

**Fig. 2:**
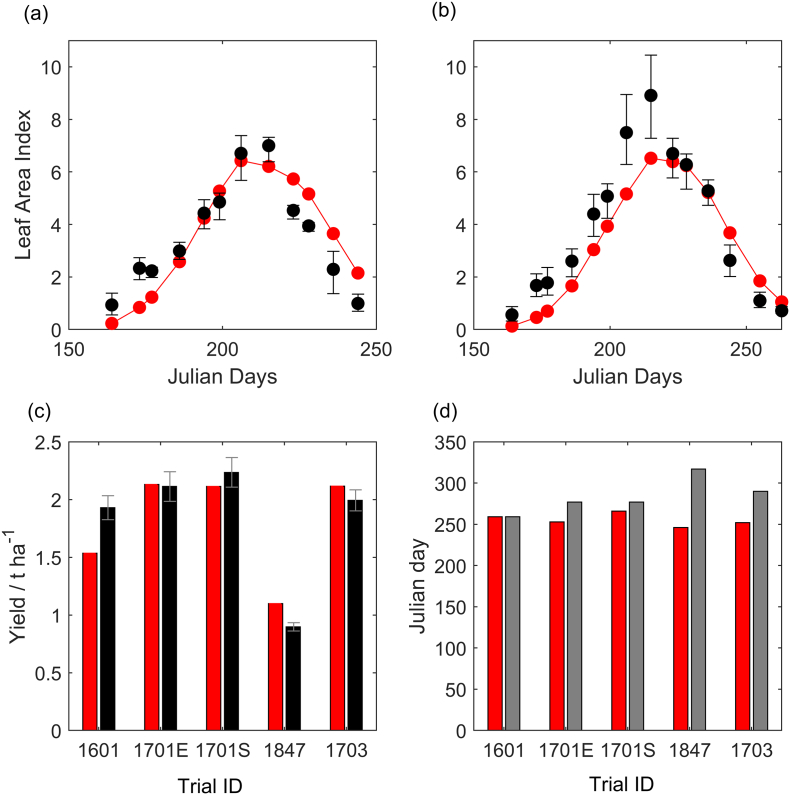
Modelled (red) and measured (black) (a) leaf area index for early sown soybean in experiment 1701. The bars show the range of observations from four replicates. (b) Leaf area index for standard sown soybean in experiment 1701. The error bars show the range of our four observations. (c) Mean yield across experiments with standard error bars. (d) The modelled maturity date and measured harvest date (grey) which indicated an upper bound for maturity. (For interpretation of the references to colour in this figure legend, the reader is referred to the web version of this article.)

**Fig. 3:**
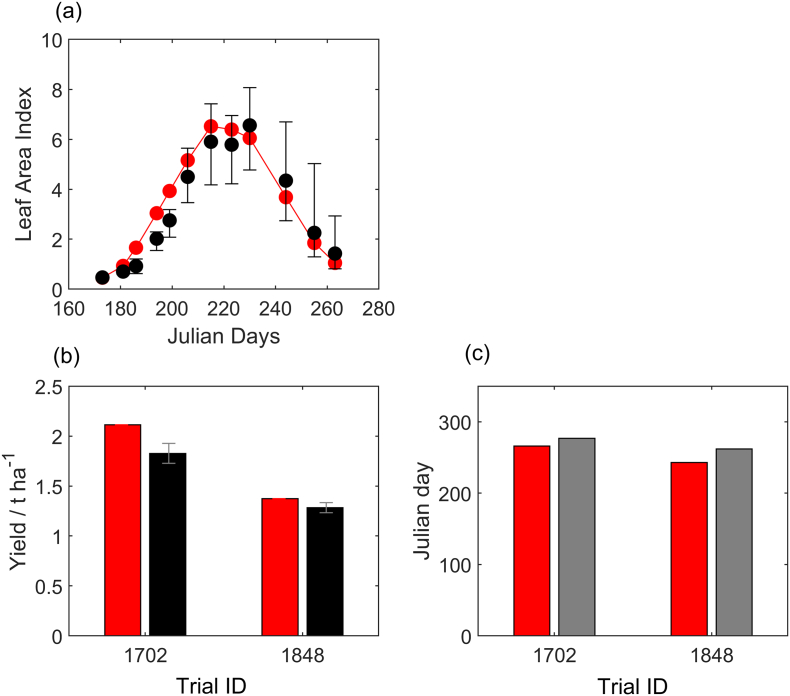
Modelled (red) and measured (black) (a) leaf area index with bars showing the range of observations from six replicates. (b) Mean yield across experiments with standard error bars. (c) The modelled maturity date and measured harvest date (grey) which indicated an upper bound for maturity. (For interpretation of the references to colour in this figure legend, the reader is referred to the web version of this article.)

The smallest RMSE between observed and predicted LAI and yield results (Fig. 2) when the LUE equals 1.6, specific leaf area equals 0.03, photo-vernal-thermal time for anthesis (DVS = 1) equals 745 and maturity is a further 400 units of photo-vernal-thermal. Validation sets performed consistently well (Fig. 3). Modelled biological N fixation, crop N uptake and N in the seed are shown in Table S9. In the model the low yields were clearly caused by water stress and lower levels of biological fixation also resulting from the unfavourable soil moisture conditions (Table S9, trials 1847 and 1848 and Table S4 and S6). Our biggest discrepancy in predicted date of maturity was site 1847, where the observed crop was harvested much later than predicted.

### 3.3 Scenario results

**Fig. 4:**
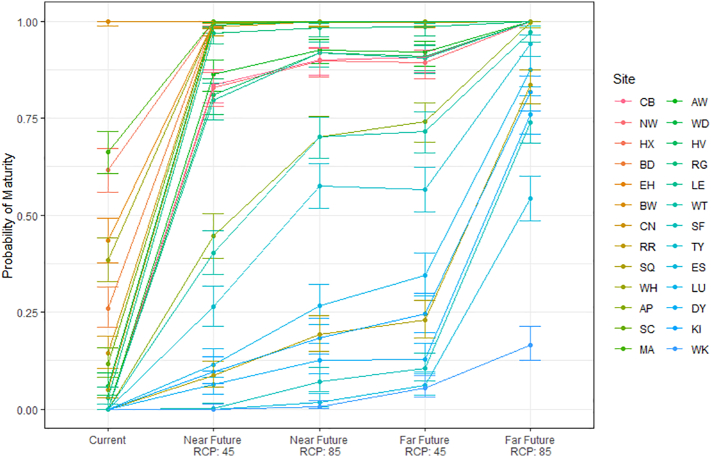
Probability of maturity calculated for each climate scenario. Error bars are the 95% Clopper-Pearson confidence intervals. Colour indicates the Site, with colour scale defined by the order of latitude (red = Southernmost site and blue = Northernmost site). (For interpretation of the references to colour in this figure legend, the reader is referred to the web version of this article.)

**Fig. 5:**
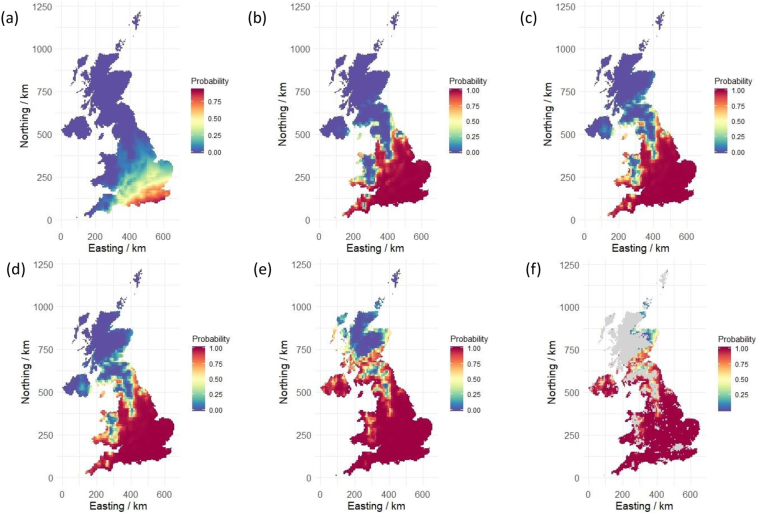
Predictions of the probability that soybean crops will mature for (a) current weather, (b) near-future-RCP4.5, (c) near-future-RCP8.5, (d) far-future-RCP4.5, (e) far-future-RCP8.5, (f) far-future-RCP8.5 with areas where crops are not currently grown masked out (grey).

Under the current climate scenario only a single site (BD) could guarantee maturity by 1st October (95% CI: 0.988, 1.000), whilst under the most extreme climate scenario (far-future-RCP8.5) 16 sites matured 100% of the time, and only a single site (WK) matured less than half of the time (95% CI: 0.125, 0.213). In the more southerly sites, the greatest increase in probability of maturity is seen in the near-future-RCP4.5, scenario (Fig. 4). Little difference is observed between near-future-RCP8.5 versus far-future-RCP4.5 (Fig. 4). In the more northerly sites, there is a general trend of increasing the probability of maturity from near-future-RCP4.5 to far-future-RCP8.5 (Fig. 5). See Fig. S5 in the Supplementary information for the numbers of simulations that successfully matured under each climate scenario. The spatial predictions illustrate clearly that the probability of maturity increases under future climate predictions, particularly in the south (Fig. 5). See Supplementary information (Table S11, Figs. S6, S7) for the parameters of the spatial models and maps of predictions of the probability that soybean crops will mature and associated errors of prediction.

**Fig. 6:**
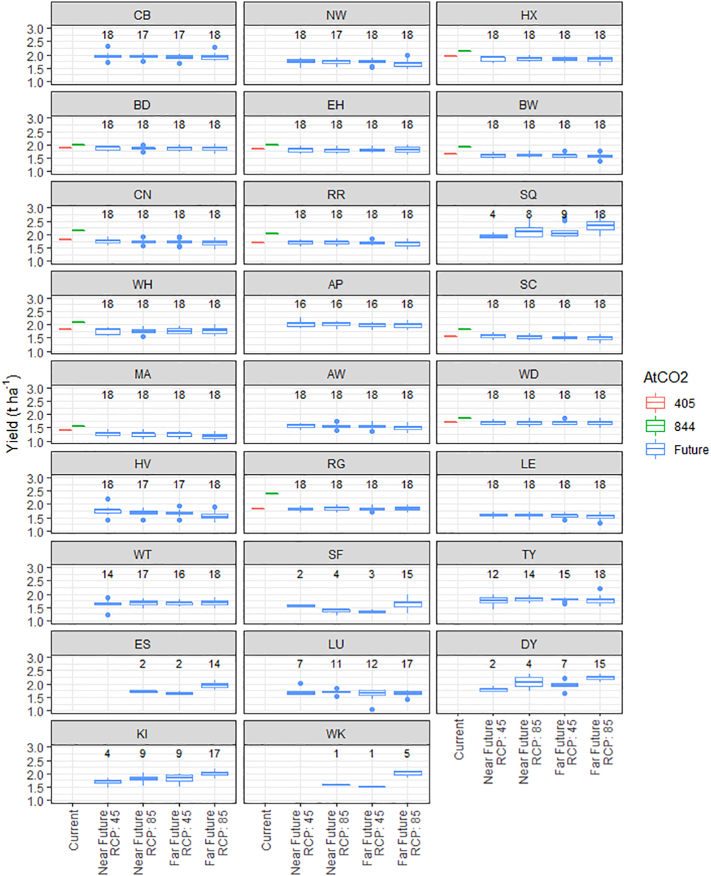
Boxplots of the expected yield under different climate scenarios at 26 locations in the UK. Values shown under current climate are the average of up to 300 individual simulations. Boxplots under future scenarios are constructed from up to 18 individual values (actual values indicated above each box in figure), one per GCM, each of which is the result of averaging over a maximum of 300 individual simulations.

**Fig. 7:**
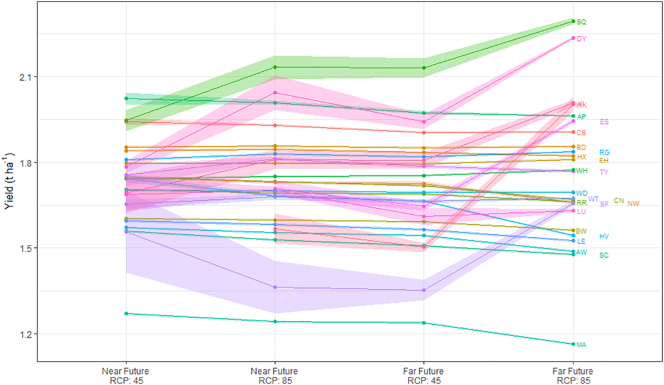
Average yield per site under each future climate scenario after having adjusted for GCM (points). Predictions are obtained by first forming the full table of predictions for all Site × GCM × RCP × Climate combinations that are present and then by averaging over GCM. The shading around each point indicates the standard errors based on marginal weights, which here reflects the number of unique GCMs for each scenario. We note that the interpolations between each point are an to aid visual interpretation but have no physical meaning.

Although future climate scenarios predict an increase in the probability of soybean maturing, the magnitude of the associated yields is less certain (Fig. 6, Supplementary information Fig. S5). Investigating the partition of variability between the different simulation scenarios (Table 5), location is the main factor for different yield predictions (marginal F_25,417__,__825_ = 7261) ranging from an average (over all climate scenarios) of 1.23 t ha^−1^ (MA) to 2.16 t ha^−1^ (SQ). It is clear that where maturity can already be reached under the current climate, substantive increases in yield are expected with increasing atmospheric CO_2_ (F_1,417__,__825_ = 339). However, given the large variation observed from different GCMs (marginal F_1,417__,__825_ = 1334), little overall effect can be observed in the 4 future climate scenarios on the predicted soybean yield. When accounting for site to site variation and the variation due to GCMs, future time period (conditional F = 903) has a larger impact on yield predictions than RCP (conditional F = 274) overall. We note that there are levels of confounding between these variables and so caution against over interpretation. The yield trends over future climate scenarios are not consistent across sites (Fig. 7). There is a slight decrease in yield at the majority of the sites as RCP changes from 4.5 to 8.5 or when period changes from 2041–2060 to 2081–2100. However, there is a small subset of sites where the yield increases substantially. In general, those sites with large predicted increases in yield (SQ, ES, KI, SF, DY, WK) are also the sites with least probability of maturing.

**Table 5:** F-statistics assessing the effect of each term on soybean yield. Marginal F-statistics are associated with including that term to the simplest possible model (respecting marginality), e.g. For a model fitting A + B + A.B, the marginal statistic for A is associated with fitting only A, the marginal statistic for B is associated with fitting only B, the marginal statistic for A.B is associated with A.B after fitting the respective main effects A and B. Conditional F-statistics are associated with including that term to the most complicated model (excluding terms to which it is marginal). E.g. for a model fitting A + B + A.B, the conditional statistic for A is associated with fitting A after accounting for B, the conditional statistic for B is associated with fitting B after accounting for A, the conditional statistic for A.B is associated with fitting A.B after fitting A and B.

Term	Marginal F statistic	Conditional F statistic	ndf	ddf (full model)
Climate	368.5	1072.4	1	417825
Site	7261.4	7529.6	25	
Climate.RCP	585.6	274.8	1	
Climate.Period	118.8	903.9	1	
Climate.GCM	1334.7	1529.3	17	
Climate.AtCO2	339.2	339.2	1	
Climate.Site	8.5	8.5	10	
Climate.RCP.Period	366.0	96.6	1	
Climate.RCP.GCM	62.6	86.9	17	
Climate.Period.GCM	76.3	102.0	17	
Climate.Site.RCP	259.9	83.7	25	
Climate.Site.Period	202.5	71.5	25	
Climate.Site.GCM	46.3	40.5	400	
Climate.Site.AtCO2	3.2	3.2	10	
Climate.RCP.Period.GCM	78.6	115.9	17	
Climate.Site.RCP.Period	32.3	24.7	23	
Climate.Site.RCP.GCM	4.8	5.4	333	
Climate.Site.Period.GCM	4.6	5.4	329	
Climate.Site.RCP.Period.GCM	2.9	2.9	305	

ndf and ddf are the numerator and denominator degrees of freedom of the F statistic, respectively.

## 4 Discussion

Our results suggest that by 2050 soybean should be a viable crop across most of England and south Wales under both RCP scenarios. In southern England the soybean early-maturing variety parameterised in our model is predicted to be certain to mature and so it is extremely likely that varieties that mature later will also be viable. This could have implications for increased yield as the growing season would be extended. To test this further we would need to calibrate the soybean model for these different types of variety, including maturity group specific functions of the effect of daylength on development (Setiyono et al., 2007). Only after 2040 and with the RCP85 prediction does soybean appear viable for Scottish agriculture.

A number of soybean models exist in the literature (Jego et al., 2010; Sinclair et al., 2003). These range from quite complex models such as the CROPGRO-soybean model (Hoogenboom et al., 1992; Jones et al., 2003)which has successfully simulated a number of cultivars and in a range of environments including Australia and across the USA (sites ranging from Florida to Idaho), and SOYDEV (Setiyono et al., 2010; Setiyono et al., 2007; Setiyono et al., 2008) which was developed to simulate soybean development under high-yield conditions of North-Central U.S. Corn Belt, to simpler models such as Sinclair-Soybean (Setiyono et al., 2010; Sinclair et al., 2003). Soybean have also been parameterised in the WOFOST (Abadi et al., 2018) and LINTUL (Corrêa, 2008) crop models for studies in Indonesia and Brazil respectively. Most parameterisations are cultivar specific, although efforts have been made to make models more parsimonious by parameterising according to maturity grouping and cultivar stem termination type (Setiyono et al., 2010). For our analysis we chose to use a more generic model to simulate our early maturing varieties, and to avoid issues of overfitting, the model parameter values were largely based on crop physiology-based values from the literature with only four parameters fitted to the data. The data provided by our trials proved a good resource to parameterise our model. In particular, data on canopy expansion (which are relatively rare) gave us confidence that the crop development was captured by the model. The calibrated model was able to reproduce the canopy expansion and decline well (Fig. 2) and accurately predicted the variation in expected yield across the seasons. This was born out in our model validation (Fig. 3). In particular, our simulation predicted the drought conditions in the soil in 2018 (as described above) and the poor yields that resulted (Table 3, trials 1847 and 1848). Our values of crop N uptake (156 kg N ha^−1^, S.I. Table S9 average of all trials) are in accordance with those reported in Bender et al. (2015), which were 164 kg N ha^−1^ for Soybean yielding 2 t ha^−1^. In our model we chose not to include an energy cost to the plant for biological N fixation. Whilst we acknowledge that any form of BNF in crops has an energy cost associated with it (Liu et al., 2011; Minchin and Witty, 2005; Vance and Heichel, 1991), Santachiara et al. (2018), found no evidence to suggest that BNF constitutes an extra energy cost to soybean crops in terms of growth or yield. This suggests that under agronomically relevant conditions this energy cost is somewhat compensated for, and that it does not substantially alter the relationship between crop biomass and crop N accumulation, particularly when yields and N uptake are relatively low (as observed in our field trials). We note however, that some models represent such C-N interactions in more detail, whereas others do not and treat N uptake more independently (see Fisher et al., 2010). Tamagno et al. (2018) list a number of mechanisms by which soybean might yield as well from BNF as it does from chemical fertilizer: increased photosynthesis, the availability of N throughout growth as opposed to dosage at a specific time and change in the nitrogen harvest index. In their experiments, however, Tamagno et al. (2018) found that at the crop level, soybean met the cost of BNF by a reduction in seed yield mediated by lower harvest index (HI), particularly in stressful environments, and a secondary contribution from reduced seed oil concentration. The soybean crops in our experiments did not receive fertilizer N and so we are unable to assess the contribution of fertilizer N compared to BNF. Given the low yields and the lack of data on the cost of BNF, we chose to disregard it in our model, which is also in line with how other soybean models have treated N fixation, uptake and partitioning. See, for example, Sinclair et al. (2003). Should yields improve through breeding or climate change, it might become necessary to revisit this part of the model and determine what mechanisms, if any, compensate for the carbon cost of N fixation.

The yields from our experiments ranged between 0.4 t ha^−1^ and 2.9 t ha^−1^ with an average of 1.7 t ha^−1^ (Table 3 and Supplementary information). These yields are slightly low within the context of global and European average yields which are reported to be closer to 2.8 t ha^−1^ and 2.08 t ha^−1^ respectively (Terzić et al., 2018). It follows that our predicted yield across the UK are generally low (for current climate average yields for a given site-year range between 0.9 and 2.0 t ha^−1^). There was no obvious spatial pattern in determining where yields were likely to be greatest under current climate, and this is likely to be because yield depends on both soil and weather (unlike phenological timing which is driven by temperature and daylength). Our predictions show that increasing CO_2_ levels will have a significant effect on yield increase (Fig. 6). However, there is a slight decrease in yield at the majority of the sites as RCP changes from 4.5 to 8.5 or when Period changes from 2041–2060 to 2081–2100. This suggests that the effects of water and heat stress may compensate for the positive effects on yield of increased CO_2_. These factors could be addressed through variety choice and breeding as explored by Semenov (2009) for wheat crops in the UK.

Despite the observed and predicted low yields of soybean in UK conditions the crop may still be financially viable for farmers. Besides land rental, the operational cost of production of soybeans is currently modest. Few pests or diseases have been observed meaning that no pesticides other than herbicides are needed, although it is acknowledged that growing a greater area of soybean is likely to results in greater pest and disease incidence (Engering et al., 2013; Legg, 1999). Based on the estimated price of soybean and associated variable costs a 2 t ha^−1^ crop could result in a gross margin of 468 £ ha^−1^ which is comparable to the profit margin of field beans (Nix, 2020; Soya, 2018). When the rotational benefits of soybeans (as described in the introduction) are also considered the crop is an attractive proposition for farmers. More viable, however, would be a scenario in which soybean consistently yields around 2.5–3 t ha^−1^ under UK conditions. Our experiments suggest that this is possible in principle, but will require further genetic and agronomic fine-tuning.

Hence, a key question is what are the major crop phenological or physiological constraints that need to be overcome to make soybean a competitive crop in the UK and other parts of Northern Europe nearby maritime Northern Europe that are at similar latitudes to the UK but have slightly warmer summers and so where soybean is equally or more likely to mature. Our canopy measurements showed that peak LAI values were similar to crops grown in Nebraska, USA that typically yield 4.5–5 t ha^−1^ (Setiyono et al., 2008). That is to say, canopy development and closure did not seem to play a major role in limiting the yields we observed. Setiyono et al. (2008) found that their green leaf persisted longer than ours; this may be because they irrigated their crops. It is worth noting that our experiments report on early-developing varieties chosen for the current UK climate. To our knowledge, there are no breeding programmes for soybean in the UK at this current time, which raises the question for breeders of whether it is possible to breed varieties that retain green leaf for longer than at present. In the future, the last frost day in spring is likely to occur up to one month earlier than now (data not shown). Although this does not necessarily translate into one month's earlier sowing and longer growing season, it suggests that later developing, and potentially, higher yielding varieties will become viable in the UK and other Northern European countries in coming decades. There is also need to better tailor agronomic practices of growing soybean to UK soil and climatic conditions. Tillage, row spacing, seed rate, inoculation, starter fertilizer along with the seed, other nutrient applications, or irrigation are all practices we did not study in our work, but which are likely to be critical for exploiting the attainable yield potential.

## 5 Conclusions

Model-based prediction shows that early maturing varieties of soybean can be grown in the UK at latitudes lower than approximately 52.3°, although yields are slightly less than the average for other European countries. Under climate change, the potential for successfully growing soybean increases enormously, with predictions under far-future-RCP8.5 suggesting the crop could be viable as far North as southern Scotland with site DY (latitude 57.21 and longitude −2.2) predicted to mature 76% of the time.

Yields are expected to respond positively to increases in CO_2,_ with average increases associated with CO_2_ only ranging from 9.1% (site EH) and 29.4% across sites (site RG), but this is tempered by increased water stress due to more evaporation meaning that only certain sites might see a positive effect of climate change on yield. With climate change, however, varieties that mature later will become viable in the south and this will also have positive implications on yield potential.

## CRediT authorship contribution statement

**Kevin Coleman:** Conceptualization, Methodology, Software, Validation, Formal analysis, Investigation, Data curation, Writing – original draft, Writing – review & editing, Visualization, Supervision, Project administration. **Andrew P. Whitmore:** Conceptualization, Methodology, Software, Investigation, Writing – original draft, Writing – review & editing, Supervision, Project administration, Funding acquisition. **Kirsty L. Hassall:** Methodology, Software, Formal analysis, Investigation, Data curation, Writing – original draft, Writing – review & editing, Visualization. **Ian Shield:** Conceptualization, Methodology, Validation, Investigation, Data curation, Writing – original draft, Writing – review & editing, Funding acquisition. **Mikhail A. Semenov:** Methodology, Software, Investigation, Writing – original draft, Writing – review & editing. **Achim Dobermann:** Conceptualization, Methodology, Writing – review & editing, Funding acquisition. **Yoann Bourhis:** Software, Investigation, Writing – review & editing. **Aryena Eskandary:** Software, Investigation, Writing – review & editing. **Alice E. Milne:** Conceptualization, Methodology, Software, Validation, Formal analysis, Investigation, Data curation, Writing – original draft, Writing – review & editing, Visualization, Supervision, Project administration, Funding acquisition.

## Declaration of competing interest

The authors declare that they have no known competing financial interests or personal relationships that could have appeared to influence the work reported in this paper.

## Appendix A Supplementary information

Supplementary information Image 1
