# Supplementary material for: The potential for soybean to diversify the production of plant-based protein in the UK
Source: Sci Total Environ. 2021 May 1;767:144903. doi: 10.1016/j.scitotenv.2020.144903 (PMC7938380; doi:10.1016/j.scitotenv.2020.144903)
Supplement: Supplementary file 1 — Supplementary information [file mmc1.pdf]

The potential for soybean to diversify the production of plant-based protein in the UK

Kevin Coleman[a*], Andrew P. Whitmore[a], Kirsty L. Hassall[b], Ian Shield[a], Mikhail A. Semenov[c], Achim Dobermann[d], Yoann Bourhis[a], Aryena Eskandary[a], Alice E. Milne[a]

[a] *Sustainable Agriculture Sciences department, Rothamsted Research, Harpenden, Hertfordshire, AL5 2JQ, UK*

[b] *Computational and Analytical Sciences department, Rothamsted Research, Harpenden, Hertfordshire, AL5 2JQ, UK*

[c] *Plant Sciences department, Rothamsted Research, Harpenden, Hertfordshire, AL5 2JQ, UK*

[d] *Directorate, Rothamsted Research, Harpenden, Hertfordshire. AL5 2JQ. UK. Present address: International Fertilizer Association, 75116 Paris France*

*Corresponding author

E-mail address kevin.coleman@rothamsted.ac.uk

Analysis of Field Trials

Soya has been grown in the UK for a number of years, but using varieties bred in Eastern Europe (Belarus and Ukraine) which are late to harvest. Field trials were undertaken to test the potential of the recent early maturity soya bean breeding lines and varieties from North American breeders as crops for the UK. There were six trials over 3 years (2016-2018) investigating both different varieties and different agronomic practice.

Table S1: Details of the six trials at Harpenden (H) and Brooms Barn (B). The trials used as our validation set are marked by *.

Trial ID	Year	Site	Field Name	Number of varieties grown	Sowing Dates	Seed Rate /seeds m^{-2}	Harvested†
1601	2016	H	Great Field 4	9	27th April	45	22nd September
1701	2017	H	Great Knott 3	2	3rd and 28th April	60 and 90††	4th October
1847	2018	H	Great Knott 3	6	25th April	60	13th November
1703	2017	B	Dun Holme	12	27th April	60	17th October
1702	2017*	H	Fosters	12	28th April	60	4th October
1848	2018*	B	Marl Pit	6	10th May	60	19th September

† Some trials were harvested over a number of days for practical reasons and the date given is the earliest of the recorded dates.

†† Two seed rates were explored in trial 1701. We only include the standard seed rate in the model calibration and so these data are not presented in the main text.

Weather data for the Field Trials

The met data we used for the Harpenden (Rothamsted) and Brooms Barn sites is freely available from the electronic Rothamsted Archive (e-RA) (http://www.era.rothamsted.ac.uk/) please contact the e-RA curators (era@rothamsted.ac.uk) for more information.

Table S2: Weather data (temperature and rainfall) from Harpenden 2016

	Min. Temperature	Max. Temperature	Ave. Temperature*		Rainfall*	
	C	C	C		mm	
January	-5.3	13.7	4.9	(1)	92.2	(22.2)
February	-3.8	13.6	4.8	(0.9)	46.7	(-3.4)
March	-3.0	13.4	5.5	(-0.8)	84.3	(33.5)
April	-1.7	16.2	7.7	(-0.6)	61.8	(6.7)
May	-1.1	25.1	12.6	(1.2)	39.3	(-15.4)
June	8.0	23.9	15.1	(0.7)	84.6	(31.3)
July	8.4	31.0	17.5	(0.7)	27.0	(-22.9)
August	8.6	31.2	17.8	(1.1)	30.1	(-33.6)
September	6.4	30.8	16.3	(2.2)	70.1	(12.5)
October	3.3	17.7	10.8	(0.2)	30.0	(-51.7)
November	-5.1	14.3	5.7	(-1.1)	85.6	(9)
December	-3.9	13.2	5.4	(1.1)	26.0	(-43.5)

*Departure from 30-year (1981-2010) means in brackets

Table S3: Weather data (temperature and rainfall) from Harpenden 2017

	Min. Temperature	Max. Temperature	Ave. Temperature*		Rainfall*	
	C	C	C		mm	
January	-6.0	10.0	3.1	(-0.9)	70.1	(0.1)
February	-1.8	16.0	5.9	(1.9)	38.7	(-11.4)
March	1.1	20.3	8.8	(2.5)	40.5	(-10.3)
April	-0.8	23.9	9.0	(0.7)	12.0	(-43.1)
May	-0.3	24.6	13.2	(1.7)	70.1	(15.4)
June	7.3	31.3	16.8	(2.3)	38.8	(-14.5)
July	10.8	29.8	17.5	(0.7)	73.8	(23.9)
August	6.5	26.1	16.0	(-0.7)	66.5	(2.8)
September	4.5	21.4	13.6	(-0.6)	86.8	(29.2)
October	1.9	21.1	12.3	(1.7)	31.1	(-50.6)
November	-2.3	14.4	6.6	(-0.2)	53.2	(-23.4)
December	-6.3	12.8	4.5	(0.3)	110.7	(41.2)

*Departure from 30-year (1981-2010) means in brackets

Table S4: Weather data (temperature and rainfall) from Harpenden 2018

	Min. Temperature	Max. Temperature	Ave. Temperature*		Rainfall*	
	C	C	C		mm	
January	-2.3	12.6	5.2	(1.2)	76.1	(6.1)
February	-5.8	10.1	2.3	(-1.7)	48.5	(-1.6)
March	-6.8	13.0	4.9	(-1.4)	78.3	(27.5)
April	1.9	26.7	10.3	(2)	75.0	(19.9)
May	1.2	25.6	13.3	(1.8)	61.9	(7.2)
June	6.3	27.3	16.2	(1.8)	3.5	(-49.8)
July	10.9	32.4	19.9	(3.1)	15.1	(-34.8)
August	7.0	30.6	17.4	(0.7)	64.0	(0.3)
September	2.0	24.0	14.1	(0)	51.0	(-6.6)
October	-2.6	22.8	10.9	(0.3)	71.0	(-10.7)
November	-1.9	14.8	7.9	(1.1)	63.8	(-12.8)
December	-2.1	13.7	6.5	(2.2)	75.0	(5.5)

*Departure from 30-year (1981-2010) means in brackets

Table S5: Weather data (temperature and rainfall) from Brooms Barn 2017

	Min. Temperature	Max. Temperature	Ave. Temperature*		Rainfall*	
	C	C	C		mm	
January	-4.5	9.6	3.3	(-0.8)	45.1	(-5.4)
February	-0.8	17.1	6.1	(2)	43.6	(4)
March	1.0	21.0	9.0	(2.5)	34.5	(-11.1)
April	-1.5	24.3	9.5	(0.8)	13.7	(-30.6)
May	1.7	25.0	13.6	(1.7)	67.5	(15)
June	8.1	30.6	17.2	(2.5)	93.7	(36.2)
July	9.2	26.7	17.6	(0.3)	86.2	(34)
August	8.2	27.5	17.0	(-0.2)	70.9	(8.3)
September	5.2	21.3	13.7	(-0.9)	80.2	(27.8)
October	1.4	21.7	12.4	(1.3)	19.8	(-42.4)
November	-1.6	14.7	6.5	(-0.6)	39.2	(-19.1)
December	-3.6	12.0	4.0	(-0.4)	86.0	(32.7)

*Departure from 30-year (1981-2010) means in brackets

Table S6 Weather data (temperature and rainfall) from Brooms Barn 2018

	Min. Temperature	Max. Temperature	Ave. Temperature*		Rainfall*	
	C	C	C		mm	
January	-2.6	12.9	4.8	(0.7)	66.3	(15.8)
February	-7.2	9.6	2.4	(-1.8)	41.4	(1.8)
March	-6.6	13.0	4.8	(-1.7)	78.7	(33.1)
April	2.5	26.9	10.3	(1.6)	68.9	(24.6)
May	2.4	26.7	14.0	(2.1)	27.8	(-24.7)
June	6.2	29.3	17.0	(2.2)	1.6	(-55.9)
July	9.4	34.5	20.7	(3.4)	18.6	(-33.6)
August	7.3	32.4	18.4	(1.1)	69.4	(6.8)
September	2.9	25.9	15.0	(0.4)	31.2	(-21.2)
October	-1.3	25.3	11.6	(0.5)	60.2	(-2)
November	-2.2	15.9	8.3	(1.2)	48.4	(-9.9)
December	-0.3	14.3	6.9	(2.5)	59.0	(5.7)

*Departure from 30-year (1981-2010) means in brackets

Table S7: Overview of field trials

Experiment	Statistical Design	Treatments	Site	Analysis
1601	Randomized complete block design (RCBD)	Variety (9)	Harpenden	ANOVA
1701	Split-plot	Variety (2)<br>Sow date (2)<br>Seed rate (2)	Harpenden	ANOVA
1702	Row-Col	Variety (12)	Harpenden	REML
1703	Row-Col	Variety (11 + 1 missing)	Brooms Barn	REML
1847	RCBD	Variety (6)	Harpenden	ANOVA
1848	RCBD	Variety (6)	Brooms Barn	ANOVA

In all experiments, varietal performance differed significantly (Table S1). Across the 2016 and 2017 variety trials the highest yielding cultivars gave moderate yields with the means over replicate plots having maximum of 2.72, 2.34 and 2.61 t h^{-1}. Yields in 2018 were substantially lower at a maximum of 1.08 and 1.61 t h^{-1} due to the exceptionally dry weather.

Table S8: Yield analysis from all field trials. LSD calculated either exactly from the ANOVA or as the average over the pairwise LSDs from REML. Similarly, F-statistics are exact (as obtained from ANOVA) or the Kenward-Roger approximate F-statistics from REML. See Table S7.

Exp. Code	Variety	Maturity group	Mean	SE	LSD*	df	F-statistic	P-value
1601	Canada 1	000	1.61	0.08162	0.2447	16	$F_{8,16}$=44.87	p<0.001
	Canada 2	00	1.672	0.08162				
	Canada 3	00	1.883	0.08162				
	Canada 4	000	1.413	0.08162				
	Canada 5	000	1.829	0.08162				
	Canada 6	000	1.17	0.08162				
	USA 1	0.0	2.721	0.08162				
	USA 2	0.0	2.608	0.08162				
	USA 3	0.4	2.459	0.08162				
1702	Canada 1	000	1.183	0.1162	0.3223	8	$F_{10,8}$=16.11	p<0.001
	Canada 2	00	1.568	0.1189				
	Canada 3	00	1.511	0.1164				
	Canada 4	000	2.338	0.1163				
	Canada 5	000	2.303	0.1159				
	Canada 6	000	2.059	0.1165				
	USA 1	0.0	1.661	0.1189				
	USA 2	0.0	1.264	0.1165				
	USA 3	0.4	1.594	0.1164				
	USA 4	00.9	1.904	0.1159				
	USA 5	0.007	1.476	0.1189				
1703	Canada 1	000	1.648	0.2181	0.7216	13	$F_{11,13}$=2.72	p=0.043
	Canada 2	00	1.655	0.2183				
	Canada 3	00	2.151	0.2195				
	Canada 4	000	2.299	0.2188				
	Canada 5	000	1.857	0.2181				
	Canada 6	000	2.481	0.2184				
	USA 1	0.0	1.932	0.2611				
	USA 2	0.0	2.33	0.2618				
	USA 3	0.4	1.656	0.2181				
	USA 4	00.9	2.605	0.2184				
	USA 5	00.7	1.913	0.2183				
	USA 6	00.8	1.187	0.2546				
1847	Alaska	00	0.6843	0.06037	0.1781	20	$F_{5,20}$=5.86	p=0.002
	Anser	000	1.0835	0.06037				

	Gallec*	000	0.8822	0.06037				
	Korus	00.9	0.8863	0.06037				
	Obelix*	000	1.0364	0.06037				
	USA 4	00.9	0.8132	0.06037				
1848	Alaska	00	0.913	0.06504	0.1919	20	$F_{5,20}$=15.07	p<0.001
	Anser	000	1.399	0.06504				
	Gallec*	000	1.609	0.06504				
	Korus	00.9	1.306	0.06504				
	Obelix*	000	1.403	0.06504				
	USA 4	00.9	1.065	0.06504				

*European varieties.

Analysis of the varying agronomic practice in trial 1701 showed the largest differences in yield were associated with differences in variety $F_{1,17}$=178.20, p<0.001. The highest yielding cultivar (averaged over the different seed rates and sowing dates) gave 2.57 t h^{-1}. Significant differences in yield were found due to the early/late sowing dates $F_{1,3}$=24.15, p=0.016 with late drilling yielding an average of 0.18 t h^{-1} more. Significant differences in yield were found the due to the high/low seed rates $F_{1,17}$=15.04, p=0.001, with the higher seed rate yielding an average of 0.18 t h^{-1} more. Interactions were not found to be significant.

Cultivar performance was found to be inconsistent both across years and sites. Combining the yields from both 1702 and 1703, (Figure S1) and also 1847 and 1848 (Figure S2), we see that yields are generally higher at Brooms Barn. However, individual varieties perform inconsistently across the two sites. In 2017, Canada 4,5, and 6 outperform all other varieties in Harpenden and yet are not particularly remarkable at Brooms Barn. Conversely USA 2 and 4 performed well at Brooms Barn but only USA 4 demonstrated good performance at Harpenden. It is notable that USA 1-3 performed well in 2016 (Table S8) and was greater than expected in 2017 (at Harpenden site). Conversely, Canada 4 and 6 performed well in 2017 compared to 2016, where seed quality and seed rates were an issue.

In 2018, varieties performed more consistently across the two sites, albeit at much lower yields and with greater site differences, with Alaska and USA 4 performing relatively poorly, whilst Anser and Obelix doing better.

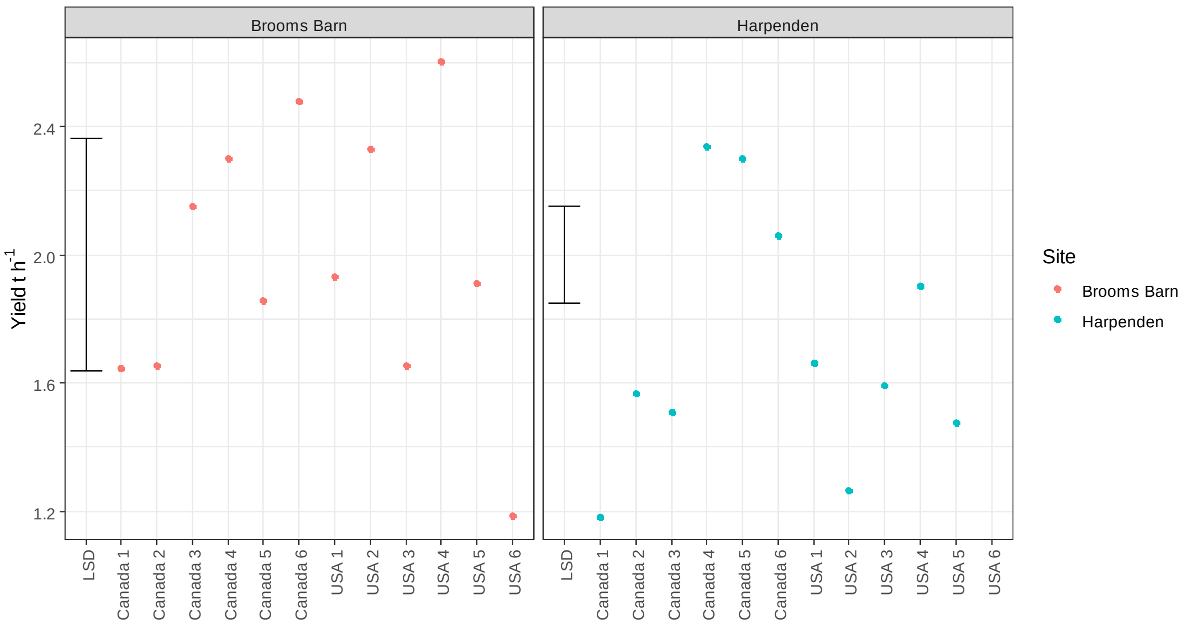

Fig S1. Predicted yields for all cultivars grown in 2017 at Brooms Barn and Harpenden. Error bar shown is average LSD for each site from a combined analysis.

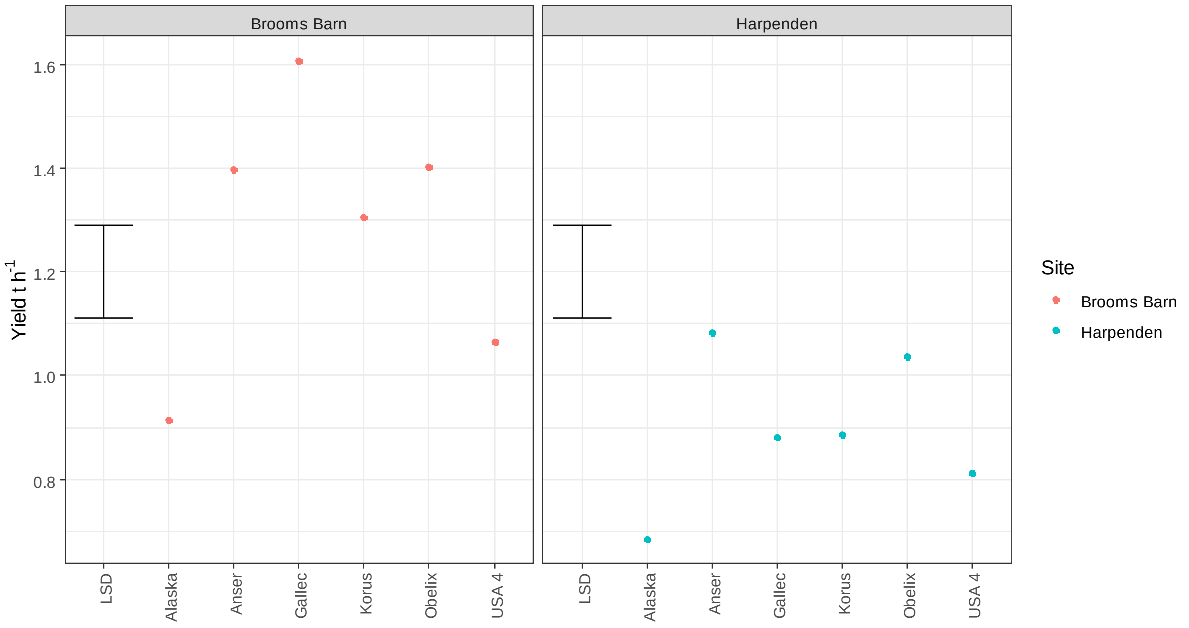

Fig S2. Predicted yields for all cultivars grown in 2018 at Brooms Barn and Harpenden. Error bar shown is average LSD for each site from a combined analysis.

USA 4 was the only variety to be grown both different sites and across different years. Analysing these yields only, we see that the effect due to year (2017 vs 2018) is by far the largest source of variation $F_{1,12}$=182.96, $p < 0.001$. However, differences can be observed at the two sites $F_{1,12}$=18.81, $p < 0.001$ and that these differences have a marginal interaction over time $F_{1,12}$=4.80, p=0.049.

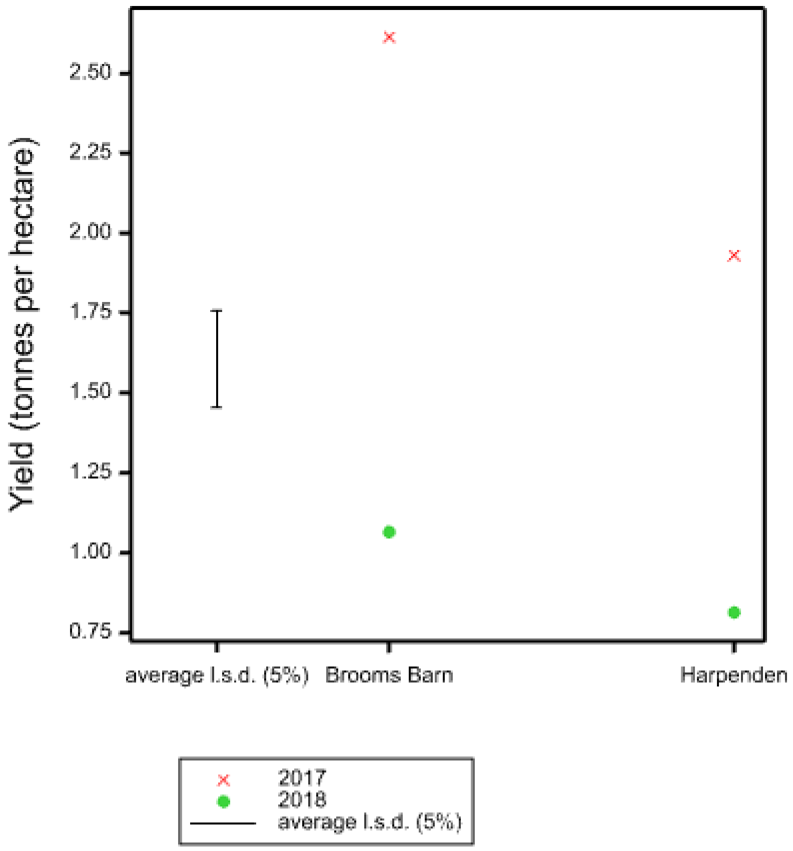

Fig S3. Yield of USA-4 grown in 2017 and 2018 at both Harpenden and Brooms Barn.

Despite the inconsistent yield performance, seed nitrogen appears to be more stable across sites (Figure S4). This was only available for the 2017 experiments where it can be seen that although there is a significant variety by site interaction, the general trends appear consistent with Canada 1-3 having highest seed N content, with USA 1,2 and 5 also having relatively higher levels.

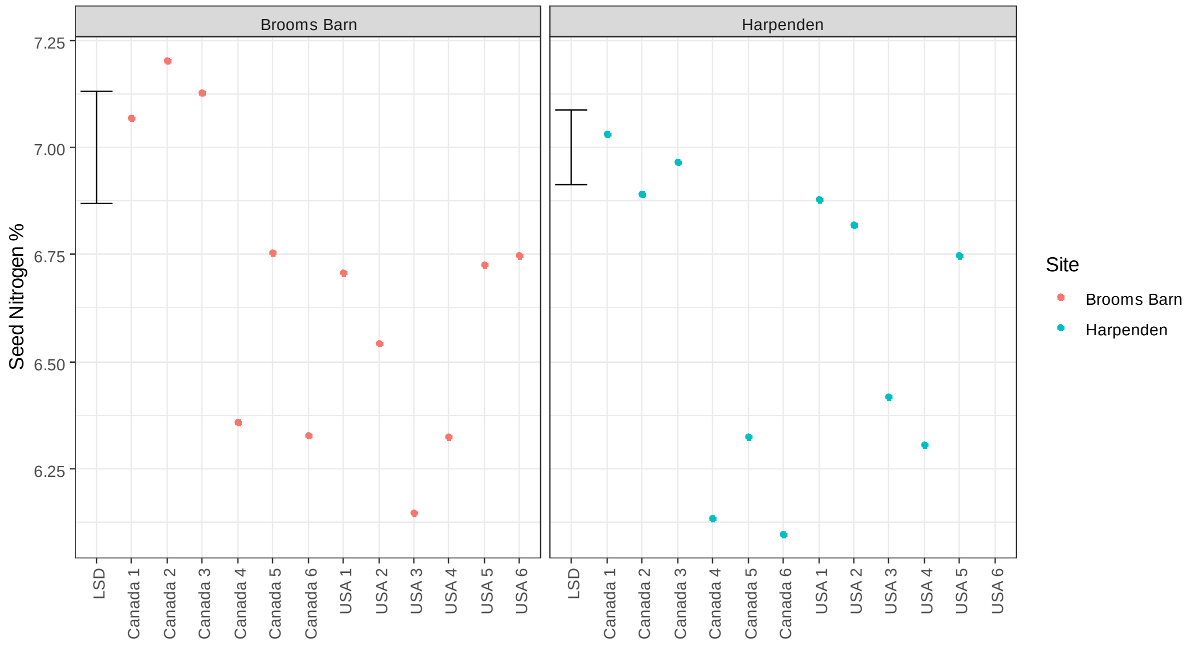

Fig S4. Predicted seed nitrogen content (%) for all cultivars grown in 2017 at Brooms Barn and Harpenden. Error bar shown is average LSD for each site from a combined analysis.

Table S9

Modelled biological N fixation, crop N uptake and N in the seed

				kg N ha-1		
Trial id	Sowing time	Year	Location	BNF	Crop N uptake	Seed N
1601	Standard	2016	H	34.3	140.4	96.9
1701	Early	2017	H	74.6	181.9	134.6
1701	Standard	2017	H	72.7	180.5	133.5
1703	Standard	2017	B	69.7	180.6	133.7
1847	Standard	2018	H	8.7	112.6	69.4
1702*	Standard	2017	H	72.7	180.5	133.5
1848*	Standard	2018	B	9.3	120.3	86.9

Predictions

Baseline weather

Table S10: Temperature and rainfall for baseline weather (1st April – 30th September)[1]

Site	Acronym	Lat	Long	Alt	Ave. min. temp[2]	Ave. max. temp[3]	Ave temp	rainfall
					C	C	C	mm
Wick	WK	58.45	-3.09	36	-2.1	21.9	10.5	353.9
Kinloss	KI	57.65	-3.56	5	-1.8	26.8	12.0	360.8
Dyce	DY	57.21	-2.20	65	-2.0	25.9	11.6	376.0
Leuchars	LU	56.38	-2.86	10	-2.1	26.2	12.0	361.2
Eskdalemuir	ES	55.31	-3.21	242	-4.1	26.5	10.8	717.7
Tynemouth	TY	55.02	-1.42	33	0.8	25.6	12.3	320.9
Shap Fell	SF	54.50	-2.68	255	-4.3	26.2	11.1	629.4
Whitby	WT	54.48	-0.60	41	-0.8	26.7	12.5	277.6
Leeming	LE	54.30	-1.53	32	-2.0	28.6	13.0	324.4
Ringway	RG	53.36	-2.28	33	-0.4	29.0	13.5	372.0
Holyhead Valley	HV	53.25	-4.54	10	0.9	26.7	13.2	373.7
Waddington	WD	53.18	-0.52	68	-0.4	29.4	13.6	322.2
Shawbury	AW	52.79	-2.66	72	-2.6	29.3	13.1	330.9
Marham	MA	52.65	0.57	21	-1.7	30.2	13.9	319.0
Church Lawford	SC	52.36	-1.33	107	-2.4	31.1	13.7	349.5
Aberporth	AP	52.14	-4.57	133	0.9	26.5	12.6	368.8
Wattisham	WH	52.12	0.96	89	-1.4	29.6	13.8	325.9
Sennybridge	SQ	52.06	-3.61	307	-3.5	26.3	11.6	558.4
Rothamsted	RR	51.80	-0.35	128	-1.5	29.6	13.5	351.6
Cardiff	CN	51.48	-3.55	70	-0.6	28.8	13.7	427.4
Bristol	BW	51.45	-2.60	42	0.5	30.3	14.7	373.7
East Hamsted	EH	51.38	0.78	75	-3.0	31.4	13.8	304.2
Boscombe Down	BD	51.16	-1.75	126	-1.6	30.1	13.8	320.7
Herstmonceux	HX	50.89	0.32	52	-0.7	29.1	14.0	319.4
North Wyke	NW	50.77	-3.90	177	-0.6	27.5	13.0	400.2
Camborne	CB	50.22	-5.33	87	1.8	25.0	13.3	409.5

[1]: April – September summaries are shown as soya was sown in April and needed to reach maturity between the end of September

[2]: Average minimum of the 300 realisations at each site of baseline weather (April to September)

[3]: Average maximum of the 300 realisations at each site of baseline weather (April to September)

Scenario results of predicted yield

Figure S5 shows the results for all simulation runs and represents both the inter-annual variability and variability due to climate uncertainty through different GCMs. Note this is paired with Figure 6 of the main manuscript which shows the expected yield under different climate scenarios having averaged over the interannual variability.

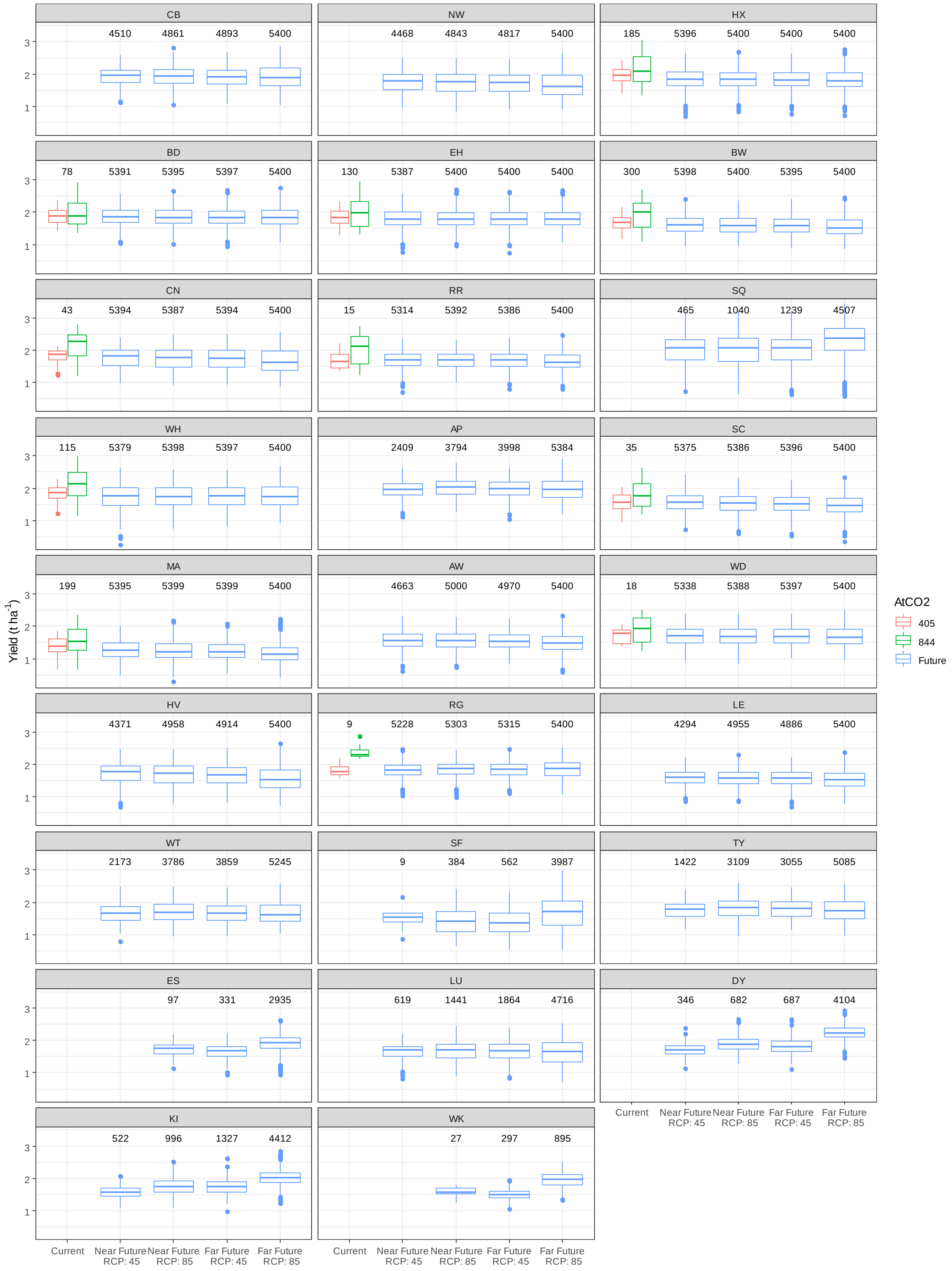

Fig S5 – Boxplots of the predicted yield for simulation runs maturing before the 1st October. Under current climate, 300 simulations were run whilst under each of the future climate scenarios, 5400 (300 x 18 GCM) simulations were run. The number of simulations resulting in maturity before 1st October are labelled above each box.

Spatial Predictions of Maturity

There are too few locations in the data set for spatial prediction by kriging. The key determinants for maturity in the model are temperature and day length, which are affected by location and elevation. Therefore, we fitted a linear model to the data with eastings (km), northings (km) and Elevation (km) as the explanatory variables as we had values for each cell of a 5km x 5km grid coving the UK from which to make predictions. For each scenario (Current, Near Future & RCP4.5, Near Future & r#CP8.5, Far Future & RCP4.5, Far Future and RCP8.5) the model took the form

$$w = c_0 + c_1x + c_2y + c_3xy + c_4x^2 + c_5y^2 + c_6z$$

where x is easting, y is northing and z is elevation and w is the logit of the probability of maturing. To avoid issues with zeros and ones we used a constant offset (5/300 for current and 5/5400 for other models) in the logit transformation. Predictions on the logit scale are shown in Fig. S6 with associated prediction errors in Fig. S7

S11: The estimated coefficients under each scenario.

Scenario	c_0	c_1	c_2	c_3	c_4	c_5	c_6	Percent variance accounted for
Current	-8.77	3.88e-2	-5.28e-4	-3.16e-5	-2.84e-5	6.16e-6	-7.60 e-03	61.2
NearLow	-2.22	5.44e-02	-1.13e-02	-1.90e-5	-4.97e-5	1.81e-6	-2.77e-02	93.2
NearHigh	-1.99	4.64e-02	-5.66e-03	-2.10e-5	-3.80e-5	-6.73e-7	-2.07e-02	94.0
Far Low	-2.15	4.55e-02	-5.31e-03	-2.34e-5	-3.59e-5	1.16e-6	-1.84e-02	91.0
Far High	7.52	9.66e-03	-1.19e-03	-5.47e-6	-1.05e-5	-8.17e-6	-1.80e-02	91.3

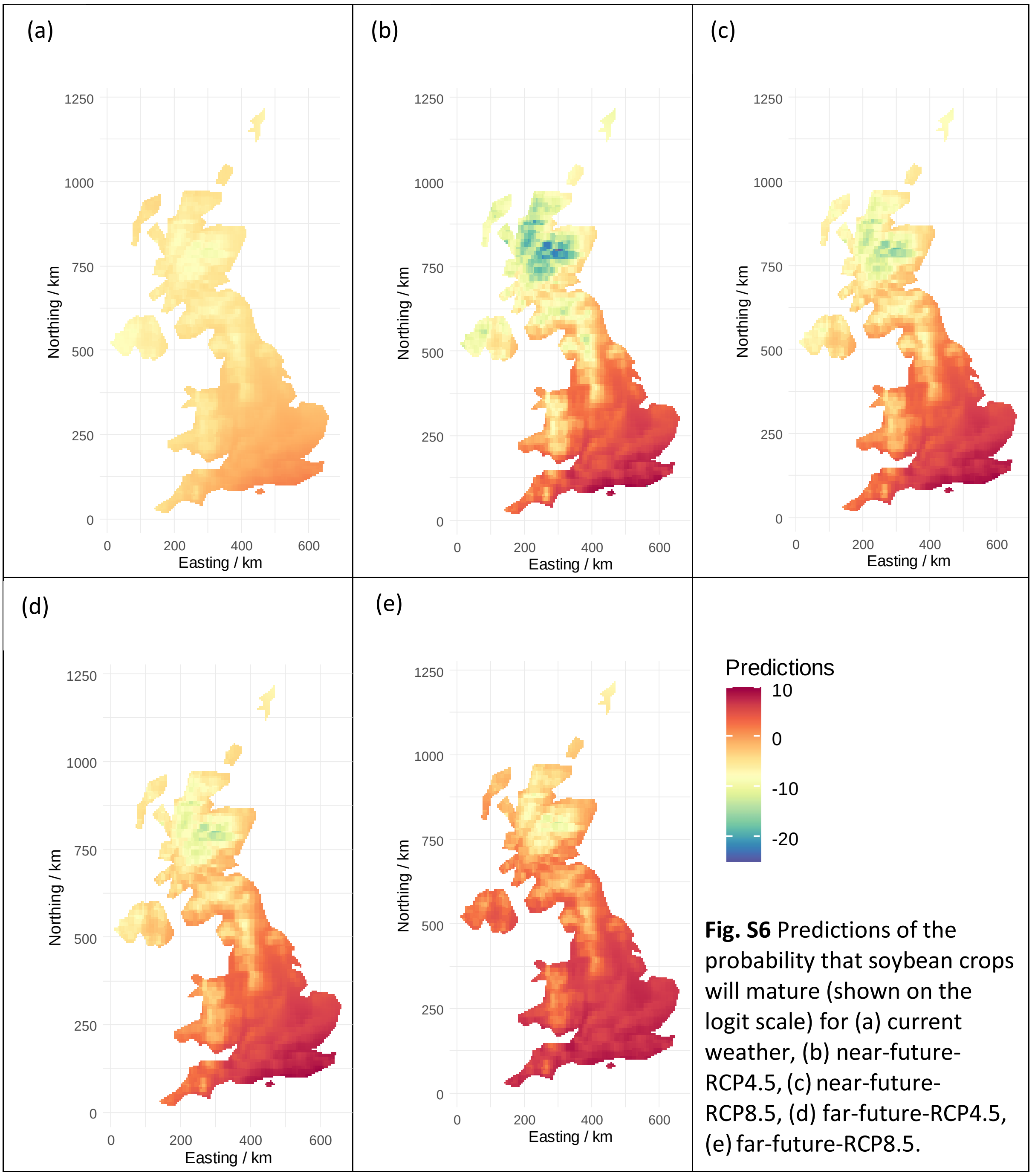

Fig. S6 Predictions of the probability that soybean crops will mature (shown on the logit scale) for (a) current weather, (b) near-future-RCP4.5, (c) near-future-RCP8.5, (d) far-future-RCP4.5, (e) far-future-RCP8.5.

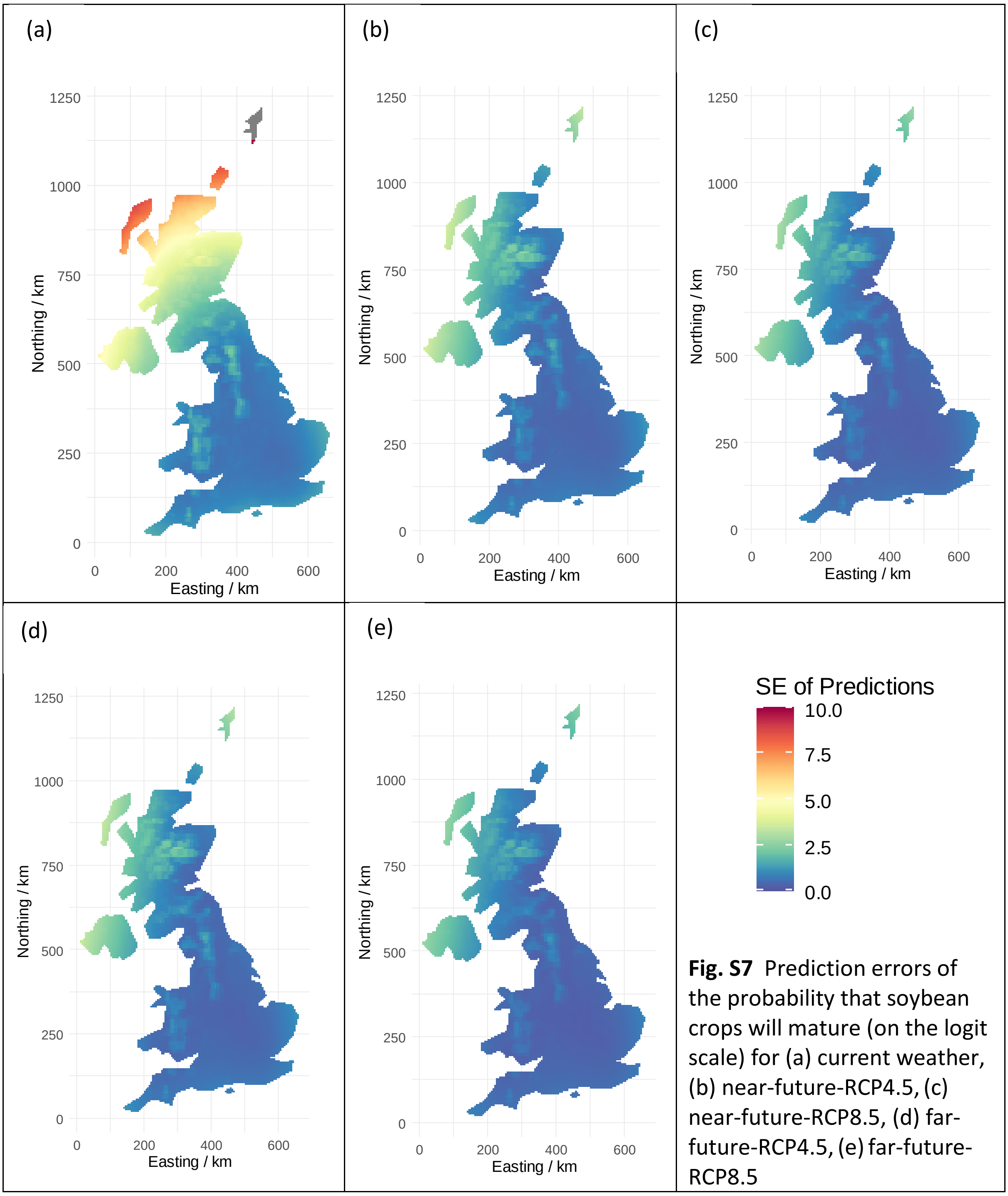

Fig. S7 Prediction errors of the probability that soybean crops will mature (on the logit scale) for (a) current weather, (b) near-future-RCP4.5, (c) near-future-RCP8.5, (d) far-future-RCP4.5, (e) far-future-RCP8.5
